# Etiology, pathological characteristics, and clinical management of black pleural effusion: A systematic review

**DOI:** 10.1097/MD.0000000000028130

**Published:** 2022-02-25

**Authors:** Zohaib Yousaf, Fateen Ata, Haseeb Chaudhary, Florian Krause, Ben Min-Woo Illigens, Timo Siepmann

**Affiliations:** a Internal Medicine, Hamad Medical Corporation, Doha, Qatar; b Division of Health Care Sciences, Center for Clinical Research and Management Education, Dresden International University, Dresden, Germany; c Department of Internal Medicine, Reading Hospital, Tower Health, PA; d Department of Internal Medicine I, University Hospital Carl Gustav Carus, Dresden, Germany; e Department of Neurology, Beth Israel Deaconess Medical Center, Harvard Medical School, Boston, MA; f Department of Neurology, University Hospital Carl Gustav Carus, Technische Universität Dresden, Dresden, Germany.

**Keywords:** black pleural effusion, pancreaticopleural fistula, pleural effusion, pleural fluid

## Abstract

**Background:**

Pleural effusion is characterized by excessive fluid collection in the pleural cavity. Black pleural effusion (BPE) is a rare entity with only limited scientific data. We aimed to review the current literature on black pleural effusion to characterize demographics, etiology, clinical presentation, pathological findings, available treatment strategies, and prognosis of this rare condition.

**Methods:**

We performed a systematic review of case reports and series and synthesized data on demographics, manifestations, management, and outcomes of patients with BPE. We searched Cochrane Library, PubMed, SCOPUS, and Google Scholar for any date until January 10, 2021. All studies (n = 31) that reported black pleural effusion in patients were added to the review. Prospective Register of Systematic Reviews registration number: CRD42020213839. Summary and descriptive analysis was performed on Jamovi version 1.2.

**Results:**

The mean age of 32 patients with BPE was 53 years, with male predominance (69%). The commonest risk factor was smoking (n = 9) followed by alcohol intake (n = 8). Dyspnea was the commonest symptom (n = 24, 75%). Pleural fluid was mostly exudative (n = 21). The commonest associated diagnosis was malignancy (n = 14), with 50% secondary to metastatic melanoma. The commonest intervention was therapeutic thoracocentesis (n = 25, 78%), and the effusion recurred in half of the cases where recurrence was reported (n = 13). In our review, we found the mortality rate to be at 20.8% (n = 20.8%). 58.3% of the patients were successfully treated and discharged home (n = 14).

**Conclusion:**

Although rare, BPE appears to be a relevant symptom as it seems to be frequently associated with modifiable risk factors and underlying malignancy. Our systematic review substantiates a vital research gap as observational research is imperative to characterize BPE further and form a basis for designing tailored diagnostic, preventive, and therapeutic strategies for BPE.

## 1 Introduction

Pleural fluid is normally present in the pleural space in a state of equilibrium, with constant secretion and reabsorption. This physiologic pleural fluid's primary function is to maintain negative intrathoracic pressure, preserving the lungs’ functionality during the inspiratory phase.^[1]^ Pleural effusion is characterized by the collection of excessive fluid in the pleural cavity. It can arise from a wide variety of etiologies that range from infectious and non-infectious inflammation, autoimmune disorders, imbalance of hydrostatic and oncotic pressures, drug reactions, iatrogenic causes, and malignancy. Pathological analysis of pleural fluid is therefore essential for diagnosing the underlying etiology. The pleural fluid appearance may vary with the underlying condition and aid in identifying the specific etiology.

Pleural effusion is commonly classified as transudative or exudative, depending on the protein and lactate dehydrogenase levels in pleural fluid compared with the serum.^[2]^ This classification is based on Light's criteria which help to narrow down the underlying disease process.^[3]^ Some distinction of the fluid characteristics can also be performed by gross inspection of its color and consistency. The appearance may vary from a transparent and clear-looking fluid to turbid purulent, hemorrhagic causing a red pleural effusion, bilious, milky secondary to chylous effusion, or rarely black colored fluid.^[4]^ The causes of black pleural effusion (BPE) range from infection, rheumatoid arthritis, pleuroperitoneal fistula, and malignancy.^[5]^ Given the paucity of literature and the rare occurrence of BPE, we aimed to perform a systematic review to characterize the manifestations, underlying diagnosis, and outcomes related to BPE to guide clinicians in managing this condition.^[5–9]^

## 2 Methods

### 2.1 Literature search

A systematic literature search was performed for articles using databases Cochrane Library, PubMed, SCOPUS, and Google Scholar for any date up to 10^th^ January 2021. Following search strategy was used: (((Pleura) OR (pleural)) AND (effusion)) AND (black). All articles in English were analyzed by 2 authors individually (ZY and FA). The protocol has been registered at the International Prospective Register of Systematic Reviews (PROSPERO; registration number: CRD42020213839).

### 2.2 Study selection

Inclusion criteria comprised case reports, case series, and other available literature describing the presentation, symptomatology, pleural fluid analysis, underlying diagnosis, treatment, and outcome of this rare condition. We considered data from adult patients >18 diagnosed with black pleural effusion as per treating physicians who have had a diagnostic or therapeutic pleurocentesis. Black effusion had to be either described or ideally, a picture of the fluid was present. We excluded any cases prior to 1950, any postmortem studies, as well as reports with any ambiguity regarding the color of effusion. All articles in English were analyzed by 2 independent reviewers (ZY and FA). Any disagreements between the 2 reviewers were resolved by consensus. The extracted articles were initially screened by their title and abstract. Full-text articles were reviewed after the initial screening. Review articles and abstracts without full texts were also excluded.

### 2.3 Data collection

Two independent reviewers (ZY and FA) extracted data on underlying diagnosis, outcome (frequencies and proportions), treatments administered (frequencies and proportions), presenting features (frequencies and proportions), biochemical and microbiological characteristics of the pleural fluid (frequency). Additional outcomes were frequency of the symptoms, signs, laboratory tests on the pleural fluid, various treatments, outcomes, and duration to the outcome. The protocol has been registered at the International Prospective Register of Systematic Reviews (PROSPERO; registration number: CRD42020213839). Two independent reviewers (ZY and FA) appraised the quality of the added cases. For the quality assessment, we used the Joanna Briggs Institute case report appraisal checklist for inclusion in systematic reviews.^[10]^ In case of any dispute among the 2 reviewers regarding the quality of an article, a third independent reviewer (HC) appraised the article separately to make a conclusion.

### 2.4 Statistical analysis

Descriptive and summary statistics were used to describe the patients’ socio-demographic parameters, with continuous variables in mean with standard deviation or median along with interquartile range as appropriate. The Shapiro-Wilk test was used to check the normality of the data. The categorical variables were reported as numbers with percentages. All data were analyzed using Jamovi version 1.2 (created in 2020, Sydney, Australia).^[11]^

## 3 Results

**Figure 1:**
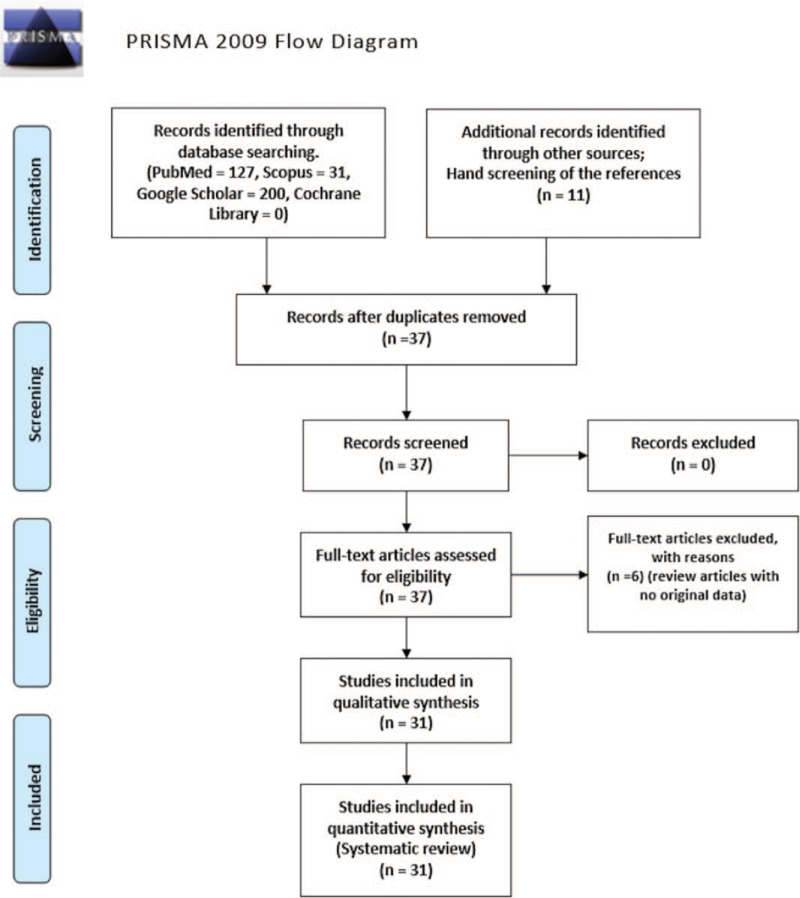
Preferred reporting items for systematic reviews and meta-analyses flow diagram of the article screening process.

Data of 32 patients with black pleural effusion patients extracted and reported from 31 eligible articles are summarized in Tables 1 and 2 concerning demographics and admission to hospital diagnosis.^[6–9,12–38]^ The flow of information through the different phases of our systematic review is depicted in Fig. 1.

**Table 1:** Baseline characteristics of patients with black pleural effusion.

Characteristics	Results
Age, yrs N = 32	53.3 ± 17.9
Gender	
Females	10 (31.3%)
Males	22 (68.8%)
Risk factors	
History of malignancy N = 28	6 (21.4%)
History of alcohol intake N = 9	8 (88.9%)
History of smoking N *=* 13	9 (69.2%)
Symptoms	
Cough N = 30	10 (33.3%)
Sputum N = 30	3 (10%)
Black sputum N = 30	2 (6.7%)
Fever N = 30	9 (30%)
Chest pain N = 31	15 (48.4%)
Dyspnea N = 32	24 (75%)
Weight loss N = 16	9 (56.3%)
Symptom duration, days	21 (12–37.5)
N = 23	
Side of effusion N *=* 32	
Right	16 (50%)
Left	10 (31.3%)
Bilateral	6 (18.8%)
Pleural fluid culture N *=* 32	6 (18.8%)
Fluid volume, mL (median) N = 11	1000 (565–1700)
Nature of effusion N *=* 25	
Exudative	21 (84%)
Transudative	3 (12%)
Purulent	1 (4%)
Frequency of concurrent infection N *=* 30	10 (33.3%)
Frequencies of therapeutic thoracocentesis N *=* 32	25 (78%)
Frequency of antibiotic use N *=* 30	10 (33.3%)
Chest tube insertion N *=* 32	11 (34.3%)
Pleurodesis N *=* 32	2 (6.3%)
Decortication N *=* 32	3 (9.4%)
Recurrence N *=* *25*	13 (52%)
Outcomes N = 24	
Death	5 (20.8%)
Palliative care	2 (8.3%)
Palliative chemotherapy	1 (4.2%)
Transfer to other facilities	1 (4.2%)
Discharge to home	14 (58.3%)
Lost to follow-up	1 (4.2%)

**Table 2:** Demographics based on admission diagnosis.

Diagnosis	Effusion type	Symptoms	Symptom duration(Median days)	Treatment	Outcomes	Recurrence
Pulmonary adenocarcinoma (N = 2)	Exudative (N = 2)	Dyspnea = 2	NA	TP = 1	Death = 1	N = 2
Hepatobiliary adenocarcinoma (N = 1)	Exudative (N = 1)	Cough = 1Chest pain = 1Dyspnea = 1Weight loss = 1	10	Abs = 1TP =1CTI = 1	Death = 1	N = 1
Mediastinal cystic teratoma (N = 1)	NA	Cough = 1Chest pain = 1Dyspnea = 1	120	TP = 1	Discharge to home = 1	N = 1
Metastatic melanoma (N* = *7)	Exudative (N* = *4)	Cough = 1Fever = 1Chest pain = 2Dyspnea = 6Weight loss = 3	25 (15.5–52.5)	Abs = 1TP = 4CTI = 2PLD = 2	Death = 1Palliative care = 2Discharge to home = 1Lost to follow-up = 1	N* = *2
Mucinous adenocarcinoma of lungs (N* = *1)	Exudative (N* = *1)	Chest pain = 1Dyspnea = 1	NA	TP =1	Palliative Chemotherapy =1	*N = *1
Pulmonary non-small cell carcinoma (N* = *1)	Exudative (N* = *1)	Cough = 1Dyspnea = 1Weight loss = 1	730	None	NA	None
Extrapleural metastatic adenocarcinoma (*N = *1)	NA	Dyspnea = 1Weight loss = 1	14	TP =1	NA	None
Pancreatic pseudocyst with fistula (N* = *9)	Exudative (N* = *8)Transudative(N* = *1)	Cough = 2Fever = 2Chest pain = 5Dyspnea = 6Abdominal pain = 1	22 (8.75 – 30)	Abs = 3TP = 9CTI = 4DC = 1	Death = 1Discharge to home = 8	N* = *4
*Aspergillus niger* empyema (N* = *2)	NA	Cough = 2Sputum = 2BlackSputum = 1Fever = 2Chest pain = 2Dyspnea = 1Weight loss = 2	74 (51 – 97)	Abs = 1Afs = 2TPI = 2CTI = 2	Transfer to other facilities =1Discharge to home = 1	None
Boerhaave hydropneumothorax (N* = *1)	Transudative(N* = *1)	NA	14	Abs = 1TP = 1CTI = 1	NA	None
Bronchopulmonary fistula (N* = *1)	Purulent(N* = *1)	Cough = 1Sputum = 1Black sputum = 1Fever = 1Chest pain = 1Weight loss = 1	28	Abs = 1TP = 1CTI = 1	Discharge to home = 1	None
Crack Cocaine (N* = *2)	Exudative (N* = *1)Transudative(N* = *1)	Cough = 1Fever = 2Dyspnea = 2	NA	Abs = 1TP = 1	NA	N* = *1
Rheumatoid pleurisy (N* = *1)	Exudative (N* = *1)	Chest pain = 1Dyspnea = 1	21	TP = 1DC = 1	Discharge to home = 1	N* = *1
Thoracic endometriosis (N* = *1)	Exudative (N* = *1)	Chest pain = 1	7	DC = 1	Discharge to home = 1	None
*Rhizopus oryza* empyema (N* = *1)	Exudative (N* = *1)	Fever = 1Dyspnea = 1	8	Abs = 1Afs = 1TP = 1	Death = 1	None

### 3.1 Demographics and risk factors

The mean age of patients was 53.3(±17.9) years. Out of the total 32 patients, 22 were (68.6%) were men, whereas 10 (31.3%) were women. If the absence or presence of a specific risk factor was explicitly reported, it was added to the analysis. Of the 32 patients with black pleural effusion, 6 (21.4%) patients had a positive malignancy history, 8 (25%) reported a positive alcohol intake history, and 9 (28.1%) had a history of nicotine consumption. Eight of these 9 smokers were men. In 19 patients, it was not reported whether patients were smokers or drank alcohol. Nine (69.2%) out of 13 patients had a positive smoking history. Smoking history was reported in 12 men, with a positive history in 8 (66.6%). In women, only 1 patient from Southeastern USA reported a smoking history (100%).^[26]^

### 3.2 Symptoms

**Figure 2:**
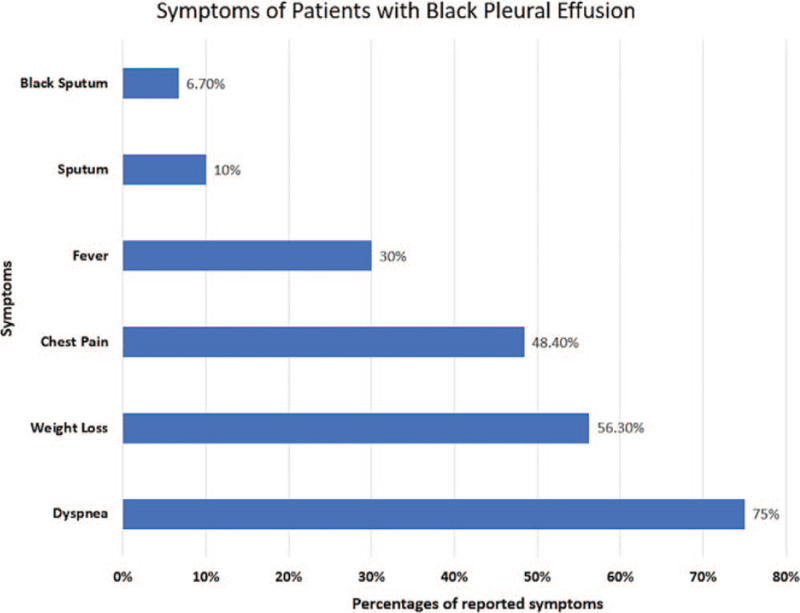
Presenting features of patients with black pleural effusion from least to most frequent.

A spectrum of pulmonary and constitutional symptoms is reported in patients with black pleural effusion. Symptoms from most common to least common include dyspnea, weight loss, chest pain, fever, sputum, and black sputum production (Fig. 2). *Aspergillus niger*-associated black pleural effusion was the only condition where the patient showed all the symptoms. All the patients who died (n = 4) had dyspnea as one of the presenting manifestations. The duration of symptoms from the onset till the diagnosis of black pleural effusion varied with the etiologies, ranging from as less as 7 days to 2 years.

### 3.3 Pleural fluid characteristics

Men had a predominantly exudative (N = 17, 89.5%) pleural effusion, whereas, in women, exudative effusion was reported in 4 (66.6%) patients according to Light criteria. The pleural fluid bacterial culture was positive in 6 patients. All positive cultures were reported in right-sided pleural effusions. Two patients had *Staphylococcus aureus* positive effusions, 1 patient had *Pseudomonas aeruginosa*, and 1 patient had *A niger* positive pleural effusion. Polymicrobial pleural effusions were reported in two patients (*P aeruginosa* + *Klebsiella pneumoniae* and *A niger* + *Serratia marcescens* + *Proteus mirabilis*). In one patient with *S aureus* infection, pleural effusion was transudative. All other bacterial pleural effusions were exudative. The median pleural fluid volume (reported in 11 patients) was 1000 mL (500–1900).

### 3.4 Etiologic characteristics

We could identify a spectrum of diagnoses associated with black pleural effusion, malignancy being the most common diagnosis (n = 14, 43.75%). Among the malignancies, metastatic melanoma was the most common (n = 7, 50%). Other malignancies include pulmonary adenocarcinoma (n = 2, 14.2%), hepatobiliary adenocarcinoma (n = 1, 7.14%), mediastinal cystic teratoma (n = 1, 7.14%), mucinous adenocarcinoma of lungs (n = 1, 7.14%), pulmonary non-small cell carcinoma (PNSCC) (n = 1, 7.14%), and extrapleural metastatic adenocarcinoma (n = 1, 7.14%). Most of the malignancies were reported in men. 85.7% (n = 12) of metastatic melanomas, 100% of adenocarcinomas, PNSCC, hepatobiliary adenocarcinomas occurred in male patients. On the contrary, women with BPE suffered from mediastinal cystic teratoma, extrapleural metastatic adenocarcinoma, and metastatic melanomas. 26.3% of the men and 12.5% of women had a history of prior malignancy. Other causes of BPE included fungal infections (n = 3), pancreaticopleural fistula (PPF) (n = 9), rheumatoid pleurisy (n = 1), crack cocaine-induced BPE (n = 2), Boerhaave hydropneumothorax (n = 1), bronchopulmonary fistula (n = 1), and thoracic endometriosis (n = 1).

### 3.5 Interventions and outcomes

The primary therapeutic interventions reported were antibiotic use (33.3%), therapeutic thoracocentesis (78%), chest tube insertion (31.3%), and decortication (9.4%). Any of the therapeutic intervention (including antibiotic use, therapeutic thoracocentesis, pleurodesis, and decortication), which was not reported, was considered not done. Pleurodesis was done in 2 patients, and both were men. A chest tube was inserted in all 6 patients with bacterial pleural effusions.

Outcomes were classified as death (in-hospital mortality), palliative care, palliative chemotherapy, discharge to home, transfer to other facilities, and loss to follow-up. One (10%) out of 10 women died. On the other hand, 4 (18.18%) out of 22 men died. Duration of symptoms to death varied from 21 days to 172 days.^[8,20,24,31,37]^ Five (71.43%) women and 9 (52.9%) men were discharged home. The recurrence rate was similar in men and women. One patient was discharged to another facility, and 1 patient was lost to follow-up.

## 4 Discussion

The major finding of our systematic review is that BPE constitutes an important research gap as previously published articles on this entity are limited to case reports, whereas the potential clinical importance of BPE is underscored by its possible association with modifiable risk factors and underlying malignancy. A previous review summarized 20 BPE cases in a narrative approach, but to date, observational data is lacking.^[39]^ There is no information in the literature with regards to the mortality burden of black pleural effusion. With the addition of a considerable number of new cases, we were able to provide more detailed results. We found a mortality rate of 20.8% in patients with BPE who had an outcome reported, with the highest number of deaths in patients with malignancy as a BPE cause. Mortality was reported to range from 2 weeks to 172 days from the symptom onset.^[8,20,24,31,37]^ We also found a recurrence rate of 52%, ranging from 24 hours to 2 years since the initial BPE drainage.^[8,12,14,18,19,22,23,25,27,29,34,35,37]^ This adds to the healthcare burden secondary to BPE. Although rare, BPE should be of particular concern as the mortality rate seems higher than the patients admitted with empyema (15%).^[40,41]^ However, we synthesized data from case reports, and therefore, our findings have to be interpreted with care. Our systematic review highlights the urgent necessity of prospective observational research to explore further the tendencies observed in our data synthesis.

### 4.1 Pathophysiology

The pathophysiology of BPE varies with the etiology. Historically, etiology has been divided into 4 main categories, i.e., malignancy, infection, hemorrhage, and other causes.^[42]^ Malignancy was by far the commonest (43.75%) cause of BPE, with a total of 14 cases. The most frequently reported cancer is metastatic melanoma (50%).^[13,16,17,20,23,35,38]^ However, BPE is also reported in primary lung malignancies.^[12,15]^ In patients with melanoma, black discoloration is a consequence of cytoplasmic melanin production by the cancerous cells.^[42]^ Patients with hemorrhagic phenomenon secondary to malignancies (such as PNSCC) or other causes (such as rheumatoid pleurisy, thoracic endometriosis) have BPE because of the hemolytic breakdown of blood. This is evident by the presence of hemosiderin-laden macrophages found in multiple case reports.^[21,36,42]^ PPF is the second most reported (28%) cause of BPE. PPF itself is a rare condition with an unknown incidence. Most cases are associated with chronic pancreatitis.^[5]^ This condition gives the black color due to the necrotic ascitic and pancreatic fluid.^[6,30]^ Another cause of BPE is infection. Three BPE cases are reported secondary to fungal infection, two with *A niger* and 1 with *Rhizopus oryzae*. The black color of the pleural effusion in *A niger* is secondary to the fungus's black-pigmented spores. Black discoloration of the effusion in *R oryzae* has been associated with necrotic processes secondary to the infection.^[9,24,26,42]^

BPE is also reported in patients with a history of crack smoking. Two such cases have been reported to date. These patients had dilated cardiomyopathy and Kaposi sarcoma, which caused pleural effusion. The pleural effusion's black discoloration was associated with the accumulation of carbonaceous particles in the pleural cavity. The same authors also examined the pleural fluid of 2 more crack smokers; however, these patients did not have black pigmentation, indicating other factors responsible for accumulating the pigment in crack smokers.^[14]^ In 1 patient admitted with alcohol and drug toxicity, activated charcoal used for therapeutic purposes caused black discoloration of pleural effusion due to esophageal perforation and tension pneumothorax.^[28]^

### 4.2 Management

Due to the diverse etiology of BPE, it is imperative to take a detailed medical and surgical history of such patients with an extensive general and respiratory physical examination. The initial investigations are the same as any other type of pleural effusion; these include imaging (preferably ultrasound if a malignant pleural effusion is suspected) followed by diagnostic (and if needed, therapeutic) thoracocentesis with pathological pleural fluid analysis.^[39,43]^ The decision to initiate antibiotics should always be guided by the suspicion or presence of an infective etiology. Pleural fluid characteristics that can strongly suggest an infective pathology include an exudative effusion with a low pH (<7.20), low glucose (<40–60 mg/dL), high C-reactive protein (>100 mg/L), and a raised white cell count (with polymorphonuclear predominance).^[44]^ Ten patients received antibiotics, but they had a suspicion or evidence of concurrent bacterial infection. The outcomes in patients who received antibiotics were comparable in the discharged and deceased groups. Therapeutic thoracocentesis, when indicated (guided by symptom severity and hemodynamic parameters), can result in an earlier clinical and radiological response. However, this is primarily dependent on the underlying etiology. We found therapeutic thoracocentesis in 78% of patients with reported management. Four out of these patients eventually died, accounting for 80% of mortalities in the patients reported having BPE.^[8,20,24,31]^ This could also indicate that this subset of patients had a larger and more aggressive BPE, which is why they required therapeutic thoracocentesis.

Decortication is usually reserved for patients with pleural effusion who have fibrothorax or the lung's entrapment.^[45]^ In our review, three patients underwent decortication. The indications for decortication were not clear. However, all 3 patients were successfully discharged. Two patients underwent pleurodesis.^[17,35]^ The underlying etiology guides targeted treatment. In patients with malignancy, the definitive treatment needs management of cancer. Likewise, patients with fungal infections require antifungals. Surgery is the definitive management of BPE secondary to PPF.^[25]^ However, some patients in our review refused surgical treatments—one of the patients who refused surgical intervention and was treated conservatively died eventually.^[31]^ One patient underwent therapeutic endoscopic stenting of the main pancreatic duct.^[18]^ Three patients underwent pancreatic drainage.^[22,30,34]^

### 4.3 Outcomes/prognosis

Various outcomes are reported in the literature among the patients with BPE, including death, successful discharge to home, transfer to other facilities, palliative care and chemotherapy, and loss of follow-up. The majority of the patients (58.3%) were discharged home. However, the patients who died were a significant proportion (20.8%) of the reported outcomes. Outcome duration was variable, and it was reported up to 2 years.^[14]^

Among the 32 patients with BPE, 14 patients were discharged successfully. Symptom duration varied from day 1 of diagnosis of BPE to 120 days. The most common symptom was chest pain (71.4%). Eight patients had a right-sided BPE, whereas 2 had bilateral and 4 had left-sided BPE. Decortication was carried out in 3 patients, and recurrence after initial drainage was observed in 6 cases.^[6,9,18,19,21,22,25,27,28,30,33,34,36,38]^ Eight (57%) among the discharged patients had BPE secondary to PPF.^[6,18,22,25,27,30,33,34]^ Only 1 patient with PPF among a total of 9 such cases died, indicating a relatively better prognosis compared with malignancy-induced black pleural effusion, with mortality rates of 11.1% and 21.4%, respectively. Antibiotic use in successfully discharged patients was comparable (35%) to the patient population who died.

Among the 5 patients who died, 3 were diagnosed with a malignancy. The underlying diagnoses were lung adenocarcinoma, hepatobiliary adenocarcinoma, metastatic melanoma, *R oryzae* empyema, and pancreatic pseudocyst with PPF. Four out of 5 patients were men with ages ranging from 46 to 86 years. Dyspnea was present in 100% of patients with mortality. Two patients had bilateral, 2 had left, and only 1 had a right-sided BPE.^[8,20,24,31,37]^ After initial drainage, recurrence of black effusion was seen in 2 patients, both with malignancy.^[8,37]^ All 5 patients had an exudative effusion. Antibiotics were given to 2 patients (40%) who had a concurrent infection.^[8,24]^ The most clinically evident difference among the discharged and deceased groups was decortication which was observed in 21.4% of discharged patients and 0% of those who died. More extensive studies powered to detect differences in outcomes based on interventions can yield essential answers in this regard.

Our review's principal strengths are its novelty and comprehensive systematic characterization of the etiology, phenotypic characteristics, and outcomes of black pleural effusion. Moreover, we identified a significant research gap and formed a basis for follow-up observational research to further define the clinical relevance of BPE and help design tailored diagnostic, preventive, and therapeutic strategies.

Our review has limitations due to the scarce literature available for BPE. First, all the data was extracted from case reports, as we could not find any other study type on the topic. Data from more extensive retrospective case–control studies could improve the validity of the results. Second, missing data in case reports such as risk factors, symptom duration, pleural fluid details, outcome limited a complete analysis.

## 5 Conclusion

Black pleural effusion is a rare phenomenon associated with diverse etiologies and pathophysiologies.

Malignancy and ruptured pancreatic pseudocyst seem to be the most common causes. Whether there is a strong clinical benefit to consider BPE as a single disease entity or merely another important parameter to describe pleural fluid is questionable. Considering the broad spectrum of etiologies and consecutive different adequate therapeutic options, classifying BPE as a condition on its own might not be warranted. However, due to the high mortality rate associated with BPE, its occurrence should be regarded as a warning sign and always lead to a prompt diagnostic and therapeutic management to decrease the morbidity and mortality of this phenomenon. Observational research is urgently needed to confirm and further assess these observations in order to design tailored diagnostic, preventive, and therapeutic strategies for BPE.

### 5.1 Ethics and dissemination

Ethical approval is not required for this systematic review as only a secondary analysis of data already available in scientific databases is conducted.

## Author contributions

**Conceptualization:** Zohaib Yousaf.

**Data curation:** Fateen Ata, Haseeb Chaudhary.

**Formal analysis:** Zohaib Yousaf.

**Funding acquisition:** Fateen Ata.

**Investigation:** Zohaib Yousaf.

**Methodology:** Zohaib Yousaf.

**Project administration:** Zohaib Yousaf.

**Supervision:** Ben Min-Woo Illigens, Timo Siepmann.

**Validation:** Zohaib Yousaf.

**Writing – original draft:** Zohaib Yousaf, Fateen Ata.

**Writing – review & editing:** Zohaib Yousaf, Fateen Ata, Haseeb Chaudhary, Florian Krause, Ben Min-Woo Illigens, Timo Siepmann.

## Correction

The funding information in this article's footnote was updated to include ‘The Qatar National Library funded the publication of this article.’
